# Memory of the vernalized state in plants including the model grass *Brachypodium distachyon*

**DOI:** 10.3389/fpls.2014.00099

**Published:** 2014-03-25

**Authors:** Daniel P. Woods, Thomas S. Ream, Richard M. Amasino

**Affiliations:** ^1^Department of Biochemistry, University of Wisconsin-MadisonMadison, WI, USA; ^2^U.S. Department of Energy–Great Lakes Bioenergy Research Center, University of Wisconsin-Madison, MadisonWI, USA; ^3^Laboratory of Genetics, University of Wisconsin-Madison, MadisonWI, USA

**Keywords:** vernalization, flowering, *Brachypodium*, epigenetics, life history

## Abstract

Plant species that have a vernalization requirement exhibit variation in the ability to “remember” winter – i.e., variation in the stability of the vernalized state. Studies in *Arabidopsis* have demonstrated that molecular memory involves changes in the chromatin state and expression of the flowering repressor *FLOWERING LOCUS C*, and have revealed that single-gene differences can have large effects on the stability of the vernalized state. In the perennial *Arabidopsis* relative *Arabis alpina*, the lack of memory of winter is critical for its perennial life history. Our studies of flowering behavior in the model grass *Brachypodium distachyon *reveal extensive variation in the vernalization requirement, and studies of a particular *Brachypodium* accession that has a qualitative requirement for both cold exposure and inductive day length to flower reveal that *Brachypodium* can exhibit a highly stable vernalized state.

## INTRODUCTION

Specific timing of flowering is an important adaptive trait that ensures flowering occurs when conditions are favorable. In many plant species, flowering takes place during a particular time of year in response to the sensing of seasonal cues, such as changing day lengths and/or temperature. One adaptation to the seasonal changes that occur in temperate climates has been the evolution of a vernalization response (Amasino, 2010; Andrés and Coupland, 2012). Vernalization is the process by which exposure to the prolonged cold of winter results in the ability to flower in the next growing season (Chouard, 1960). Cold exposure alone is typically not sufficient to induce flowering, but it often must be coupled to an additional inductive cue such as the increasing day lengths experienced during spring and summer months (Lang, 1952). Although satisfying the vernalization requirement often permits flowering after exposure to inductive cues, the key adaptive value of a vernalization requirement is that it prevents flowering in the fall season, thus ensuring that flowering does not commence as winter begins (Amasino, 2010).

## CONCEPT OF MEMORY

An interesting component of the vernalization response in some plant species is that the acquisition of the ability to flower from cold exposure is mitotically stable after plants resume active growth in warm conditions (Amasino, 2004). This “memory of winter” is readily demonstrated in species for which there is a qualitative requirement for an additional cue to flower, such as inductive photoperiods, after the cold requirement has been satisfied. One of the first studies on the stability of the vernalization state (or thermoinduced state as it was sometimes referred to) was done with a biennial strain of henbane (*Hyoscyamus niger*) that has an obligate requirement for a vernalizing cold exposure followed by long days (LD); i.e., vernalized henbane plants will not flower when grown in non-inductive short days (SD). In henbane, the vernalized state is “remembered” because vernalized plants grown in SD for long periods of time readily flower after they are shifted to inductive LD (Lang and Melchers, 1947).

## MOLECULAR BASIS OF THE MEMORY OF WINTER

A considerable amount of information regarding the molecular nature of the vernalization pathway is known from studies in the eudicot model *Arabidopsis thaliana *(Brassicaceae; Kim et al., 2009; Amasino, 2010; Song et al., 2013). The vernalization requirement is largely due to the expression of a MADS-box-containing transcription factor, *FLOWERING LOCUS C *(*FLC*), which is an effective flowering repressor (Michaels and Amasino, 1999; Sheldon et al., 1999). The level of *FLC* expression is determined by an extensive regulatory network that includes components involved in small RNA metabolism (Swiezewski et al., 2009; Heo and Sung, 2011; Song et al., 2013) as well as a protein complex that appears to have evolved specifically for *FLC* activation (the FRIGIDA complex; Choi et al., 2011; Lee and Amasino, 2013). The FLC protein prevents flowering at a molecular level by binding to the promoters of specific genes and blocking their expression (Helliwell et al., 2006; Searle et al., 2006); these genes include *FT,* which encodes the mobile “florigen” signal**in leaves as well as *SUPPRESSOR OF CONSTANS 1 (SOC1) *and* FD* in meristems. *FD, FT,* and* SOC1* encode proteins that activate a suite of floral homeotic genes such as *APETALA1 *(*AP1*) that specify floral organs (Kim et al., 2009).

Vernalization results in the silencing of *FLC* expression (Michaels and Amasino, 1999). During winter, exposure to prolonged cold results in a polycomb-like, chromatin-modifying complex initiating the modification of *FLC* chromatin, transforming it from an active euchromatic state into a stably repressed heterochromatic state that remains repressed for the rest of the life cycle (for reviews see Amasino, 2010; Andrés and Coupland, 2012; Zografos and Sung, 2012; Song et al., 2013). During cold, a unique, cold-specific polycomb component known as *VERNALIZATION INSENSITIVE 3 *(*VIN3*) is induced; the VIN3 protein is necessary for polycomb-mediated *FLC* silencing (Sung and Amasino, 2004; Wood et al., 2006; De Lucia et al., 2008). The polycomb complex adds methyl groups to histone 3 (H3) at lysine 27 (K27) residues to form trimethylated H3 (H3K27me3), and increased H3K27me3 of *FLC* chromatin appears to be one of the first chromatin changes accompanying *FLC* silencing (Bastow et al., 2004; Sung and Amasino, 2004; Finnegan and Dennis, 2007; De Lucia et al., 2008; Angel et al., 2011). Vernalization-mediated *FLC* silencing is also associated with increased lysine 9 (K9) trimethylation at H3 (H3K9me3; Sung et al., 2006a,b), and H3K9me3 appears to be required for the memory of winter at *FLC*.

The H3K27me3 modification at *FLC* spreads and persists after the cold exposure is over (Finnegan and Dennis, 2007; De Lucia et al., 2008; Angel et al., 2011), and H3K9me3 is likely to spread as well. Because the vernalized state and *FLC *chromatin modification persist through mitotic cell divisions in meristem cells after cold treatment ends, it is reasonable to think of vernalization as an environmentally induced epigenetic switch (Amasino, 2004; Schmitz and Amasino, 2007). Although *FLC* repression is maintained throughout the plant’s life cycle, the repressed state of *FLC *becomes reset to an active state in the following generation, resulting in the re-establishment of the vernalization requirement (Amasino, 2004; Schmitz and Amasino, 2007). The stable repression of *FLC* is consistent with the annual life history of *Arabidopsis* which involves the conversion of all shoot meristems to flowering which maximizes the number of progeny in a single cycle of reproduction (Amasino, 2009).

## TO HAVE OR NOT TO HAVE MEMORY

In contrast with annual plants such as *Arabidopsis*, perennials live for many years and flower repeatedly throughout their lives. For perennials to persist for multiple growth cycles, it is critical that not all of the shoot meristems become irreversibly floral; rather, some meristems need to be reserved for next season’s growth (Amasino, 2009; Turck and Coupland, 2013). Recently, aspects of the molecular basis of the perennial life history trait have been studied in *Arabis alpina*, a relative of *Arabidopsis* in the Brassicaceae (Wang et al., 2009; Bergonzi et al., 2013; Turck and Coupland, 2013). Like many accessions of *Arabidopsis*, *A. alpina* also requires vernalization in order to flower. However, unlike *Arabidopsis*, in *A. alpina* vernalization does not result in the flowering of all shoot meristems (Wang et al., 2009). Only certain meristems (those that were most actively growing before cold exposure commenced) produce flowers, whereas other meristems produce only vegetative shoots following cold exposure (Wang et al., 2009).

In* A. alpina*, not all shoot meristems become floral at least in part because vernalization is “forgotten.” In many (and probably all) Brassicaceae, a vernalization requirement results from flowering repression mediated by *FLC* or an *FLC* ortholog. In* A. alpina*, the *FLC* ortholog is known as *PERPETUAL FLOWER1* (*PEP1*; Wang et al., 2009). The *PEP1* expression pattern is similar to that of *FLC* before and during cold; specifically, it is highly expressed prior to cold exposure and is down-regulated during cold exposure. However, a key difference is that after cold exposure ends, *FLC* repression is maintained in *Arabidopsis*, whereas *PEP1* repression is forgotten: after cold exposure ends, *PEP1* mRNA levels begin to rise and eventually reach pre-vernalization levels (Wang et al., 2009). Furthermore, although the repressive chromatin mark, H3K27me3, increases during cold, it does not persist after cold exposure ends (Wang et al., 2009). Thus, the difference in the stability of chromatin modifications in *PEP1 *versus *FLC* contributes to the perennial versus annual life history trait of *A. alpina* and *Arabidopsis*. As discussed below, single-gene mutations in *Arabidopsis* can result in *PEP1*-like behavior at the *FLC* locus including a transient, cold-specific increase in H3K27me3 (Sung and Amasino, 2004).

This perennial strategy “works” in *A. alpina* because in this species (as well as in *Arabidopsis*) once flowering commences it is irreversible and no longer subject to *FLC/PEP1*-mediated repression. Some of the shoot meristems of *A. alpina *become irreversibly committed to flowering before post-vernalization *PEP1* levels rise, whereas the post-vernalization resumption of *PEP1 *expression appears to prevent flowering in other meristems thus “reserving” those meristems for the next growing season (Wang et al., 2009). Irreversible *FLC*-independent flowering results, at least in part in *Arabidopsis*, from a positive feedback loop involving the floral meristem-identity genes *LEAFY* and* APETALA1* which activate each other’s expression (Liljegren et al., 1999), and the feedback loop is not subject to *FLC *repression.

## GENETIC VARIATION FOR MEMORY

Interestingly, the stability of the vernalized, repressed state of *FLC* in *Arabidopsis* is easily perturbed through single-gene mutations (Levy et al., 2002; Sung et al., 2006a; Schmitz et al., 2008; Kim et al., 2009). For example, in *vrn1*, *prmt5*, or *lhp1* mutants, cold-mediated *FLC* repression is transient similar to that in *A. alpina – *i.e., *FLC* is repressed during cold exposure, but expression rises after plants resume growth in warmer conditions (Levy et al., 2002; Sung et al., 2006a; Schmitz et al., 2008). *VRN1*, *PRMT5*, and *LHP1 *do not encode components of the polycomb complex; rather, they encode other types of chromatin-modifying proteins involved in epigenetically “locking in” the repressed state of *FLC* such that polycomb-initiated repression becomes mitotically stable in warm conditions (Kim et al., 2009). This mitotic stability appears to require an increased level of H3K9 trimethylation at *FLC*, as well as H3K27 trimethylation (Bastow et al., 2004; Sung and Amasino, 2004; Sung et al., 2006a,b). Thus, a single-gene change can determine the difference between a memory of winter or lack thereof, and it might be expected that families of plants in addition to the Brassicaceae would contain vernalization-requiring members that had a memory of winter and members that did not depending upon the life history strategies they have evolved.

## MEMORY IN A GRASS MODEL

Vernalization-requiring species exist in many plant groups spanning angiosperm diversification (Preston and Sandve, 2013). There are additional examples of species (or varieties within a species) in which**the vernalized state is not stable in non-inductive SD conditions such as sugar beet (Caryophyllales; Margara, 1960), primrose (Myrtales; Chouard, 1960), carrot (Apiales; Bernier et al., 1981; Bernier, 1988), wheat (Poales; Evans, 1987), and *Cheiranthus* (Brassicales; Barendse, 1964). Thus, many plant species that have a vernalization requirement do not have the ability to “remember” prior cold.

We sought to determine if the small, temperate grass *Brachypodium distachyon* has the ability to “remember” prior cold. Recently, we and others have characterized natural variation in the vernalization response in many *Brachypodium* accessions, and we found considerable variation in flowering behavior, ranging from accessions that exhibit rapid flowering without prior cold exposure in inductive LD to accessions that have an obligate vernalization requirement (Schwartz et al., 2010; Ream et al., 2012, 2014; Colton-Gagnon et al., 2013; Tyler et al., 2014). Furthermore, among the accessions that have an obligate vernalization requirement, the amount of cold needed to saturate the vernalization response ranges from 2 weeks to greater than 16 weeks (Ream et al., 2014). With respect to the requirement for inductive photoperiods, none of the *Brachypodium* accessions tested flower after several months of outgrowth in SD (8-h day length) even if vernalized extensively (Ream et al., 2014) i.e., like biennial henbane, many *Brachypodium* accessions have an obligate requirement for both vernalization and inductive photoperiods in order to flower. Thus, there are *Brachypodium* accessions that can be used to investigate whether or not *Brachypodium* can remember prior cold with an experimental design similar to the classic work first done in henbane by Lang and Melchers (1947).

**FIGURE 1:**
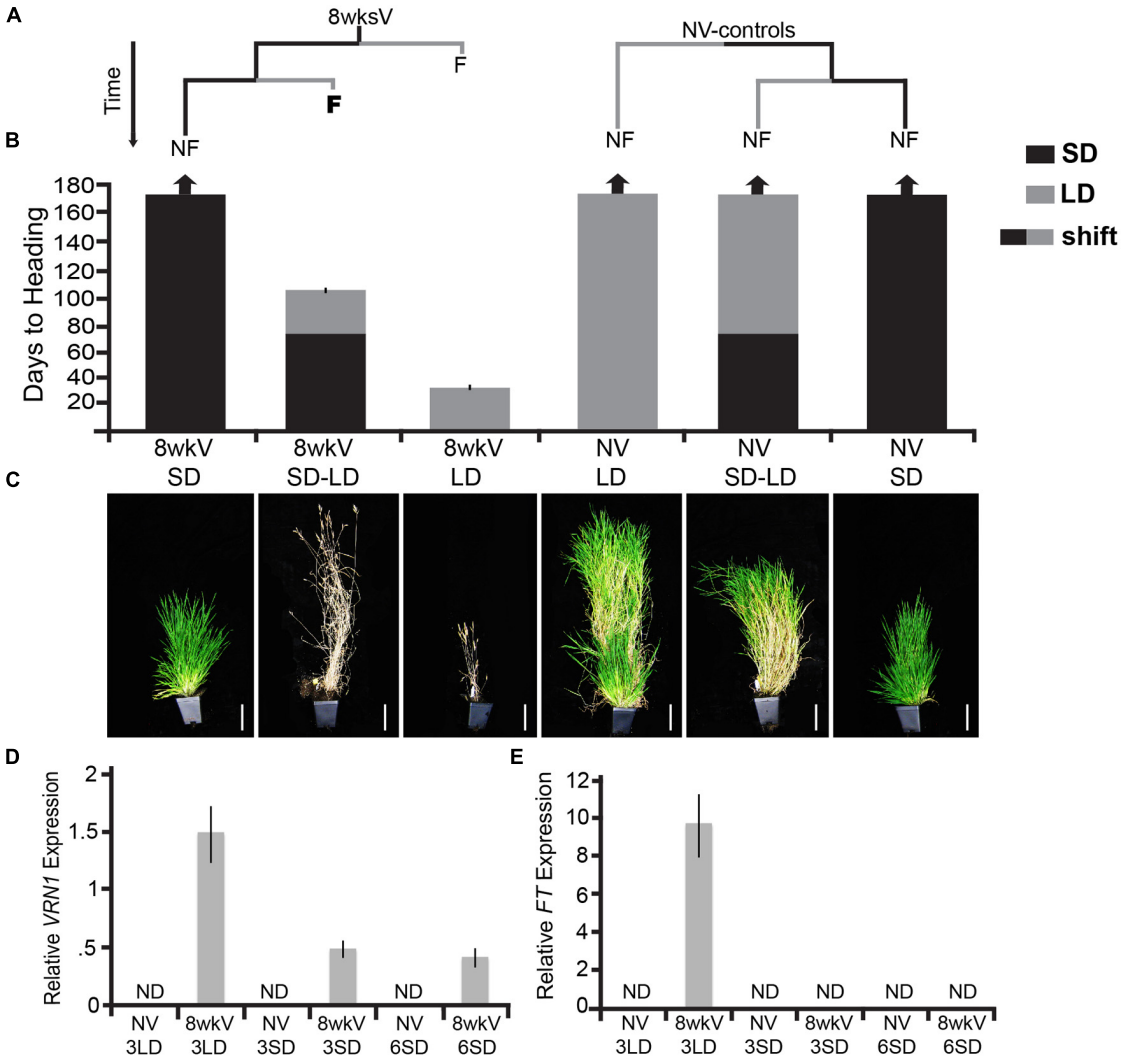
**The vernalization response is mitotically stable in *Brachypodium distachyon* (Bd29-1).** **(A)** Plants were exposed to either 8 weeks of cold to saturate the vernalization response (8wksV) or were non-vernalized (NV-controls). Both the 8wksV and NV treated plants were placed either into inductive 20-h long days (LD; indicated by the gray bar) or non-inductive 8-h short days (SD; indicated by the black bar). After 70 days, some of the plants that had been in SD were shifted into LD, and, as a control the remainder of the SD-grown plants were kept in SD for the duration of the experiment. Only vernalized plants flowered in LD indicated by the letter F (flower); plants that did not flower are indicated by NF (non-flowering). **(B)** Days to heading was measured as the number of days to first spikelet emergence as done in Ream et al. (2014). Note that time during vernalization treatment is excluded. Arrows at the top of the bar graph indicate plants that did not flower for the duration of the experiment (170 days). Black bars represent SD-grown plants; gray bars represent LD-grown plants and bars with black and gray represent those plants that were first exposed to SD for 70 days followed by a shift into LD (SD–LD). Bars represent the days to heading average of six plants (experiment was repeated with similar results). **(C) **Photographs of representative plants at the end of experiment. Plants were grown and scored as described in Ream et al. (2014). Scale bar = 9 cm. **(D,E) ***VRN1 *and *FT *expression in a newly expanded 3^rd^ leaf and 6^th^ leaf of Bd29-1. Imbibed seeds in soil were exposed to either a saturating cold treatment (5°C for 8 weeks) or no cold. At the end of cold treatment, non-vernalized and cold-treated plants were grown in SD and LD for ~3 weeks (3LD, 3SD) and SD for an additional ~6 weeks (6SD) at 22°C during the light phase and 18°C during the dark phase until plants reached the third leaf stage and the sixth leaf stage (Note vernalized 29-1 in LD had flowered by 5 weeks so did not sample 6LD). *VRN1 *and *FT *transcript levels were determined by**RT-qPCR as described in Ream et al. (2014) and normalized to *UBIQUITIN-CONJUGATING ENZYME18*. ND denotes no expression detected. Bars represent the average of four biological replicates ± standard deviation (three leaves per replicate).

To determine if the vernalized state is mitotically stable in *Brachypodium*, we grew vernalized plants in non-inductive photoperiods before shifting to inductive photoperiods (experimental design is outlined in **Figure 1A**). Briefly, we first vernalized the accession Bd29-1 as imbibed seed for 8 weeks, which is a saturating vernalization treatment (Ream et al., 2014). After the vernalization treatment, we placed the vernalized seeds (and non-vernalized controls at the same stage of development) in either non-inductive SD or inductive 20-h LD. Some of the vernalized as well as non-vernalized plants grown in SD for 70 days were then shifted to LD. The shift of the vernalized plants from SD to LD revealed that the vernalized state was robustly maintained in SD because the time to flowering after the shift to LD was the same as that for plants directly moved from cold exposure into inductive photoperiods (**Figure 1B**). Specifically, the vernalized plants directly moved into LD as well as the vernalized plants first moved into SD flowered in fewer than 30 days forming only four leaves in inductive LD prior to flowering (**Figure 1B**, leaf data not shown). This indicates that, as is the case in henbane, there are accessions of *Brachypodium* in which the vernalization response is mitotically stable and that vernalization provides only the competence to flower, given none of the SD-only controls flowered during the duration of the experiment.

There are several controls for this experiment. One is to shift non-vernalized plants from SD to LD. The reason for this control is that short days are able to substitute for vernalization in some accessions of *Brachypodium* (Schwartz et al., 2010; Ream et al., 2014) as well as in accessions of other grass species such as wheat and rye (Purvis and Gregory, 1937; Evans, 1987; Heide, 1994). However, the Bd29-1 accession was chosen for this study because growth in SD does not have any effect on the vernalization requirement (**Figure 1B**). An additional control is the growth of both non-vernalized and vernalized plants in SD or LD for the duration of the experiment. None of the SD-only control plants or the LD-non-vernalized controls flowered during the duration of the experiment [170 days; **Figure 1A**; several SD-only control plants were also dissected after 170 days of growth and all meristems were vegetative (data not shown)]. The SD-only controls demonstrate that indeed growth in SDs does not permit flowering even in vernalized plants. The LD-only controls were chosen to ensure that the robust flowering observed in the vernalized plants was indeed due to the prior vernalization treatment and not simply due to the age of the plant when shifted into inductive LD. As expected for a species with a monocarpic life history, vernalized plants grown in LD senesced rapidly after seed fill whereas non-vernalized controls were green and still actively producing leaves throughout the duration of the experiment (**Figures 1B,C**).

**FIGURE 2:**
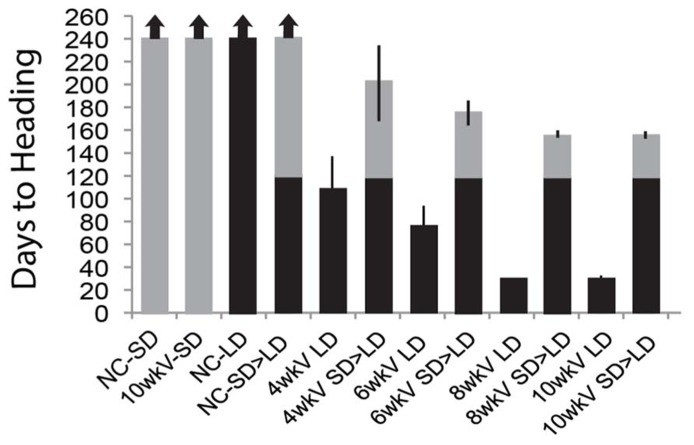
**Increasing duration of cold exposure causes increasingly rapid flowering in *Brachypodium* regardless of whether or not prolonged growth in SD separates cold exposure from inductive LD.** Plants were vernalized for 4, 6, 8, and 10 weeks then placed into either SD for 120 days followed by a shift into LD or placed directly into LD. Flowering time was measured as days to heading upon emergence of first spikelet, and the time of cold exposure (vernalization treatment) was excluded. Arrows indicate plants that did not flower at end of experiment (240 days). Bars represent the days to heading average of six plants (experiment was repeated with similar results). LD = 20-h long days (gray bars), SD = 8-h short days (black bars), SD > LD = shift after growth in SD for 120 days into LD (black and gray bars). Plants were grown and scored as described in Ream et al. (2014).

We also determined whether the quantitative aspect of the memory of vernalization is maintained in SD – i.e., is there a memory of the duration of cold exposure in *Brachypodium* when prolonged growth in SD separates cold exposure from a shift into inductive LD? Accordingly, imbibed seeds of Bd29-1 were exposed to varying lengths of cold (4, 6, 8, and 10 weeks), and then transferred to SD for 120 days prior to transfer to inductive LD (**Figure 2**; note this “memory test” is longer than the 70-day SD treatment presented in **Figure 1**). Cold exposures of 4 and 6 weeks are sub-saturating for vernalization in Bd29-1 (Ream et al., 2014). In this study, controls were similar to those presented in **Figure 1**: vernalized plants did not flower in SD and growth in continuous LD without prior vernalization also did not result in flowering illustrating the obligate nature of the vernalization requirement. A key control was the transfer of plants directly to inductive LD after cold exposure, which enabled a comparison of the efficacy of different durations of cold exposure with and without an interlude between cold exposure and photoperiodic induction of flowering. Even sub-saturating durations of cold exposure were effectively “remembered” during this long SD interlude as shown by the similar time to flowering after exposure to LD commenced in the plants exposed to 4 or 6 weeks of cold and then shifted immediately or after 120 days to LD (**Figure 2**). Saturating cold exposures of 8 and 10 weeks were also fully “remembered” during the 120-day SD treatment (**Figure 2**).

Information about the vernalization systems in Pooideae has largely been derived from studies of existing allelic variation in wheat and barley. Such studies are consistent with a flowering model in which three genes, *VERNALIZATION1 *(*VRN1*), *VERNALIZATION2 *(*VRN2*), and *VERNALIZATION3 *(*VRN3)* form a regulatory loop**in leaves that responds to vernalization and photoperiod (Greenup et al., 2009; Distelfeld and Dubcovsky, 2010). *VRN3 *is orthologous to *FT *(Yan et al., 2006) and hereafter will be referred to as *FT. *Prior to cold exposure, *VRN2 *represses *FT* expression and thus prevents flowering, whereas during and after cold *VRN2* expression decreases thus permitting flowering (Yan et al., 2004; Sasani et al., 2009; Distelfeld and Dubcovsky, 2010). Therefore, *VRN2 *occupies a position in the flowering “circuitry” analogous to that of *FLC *(both are inhibitors of flowering that are repressed by cold), although *VRN2 *encodes a protein not related to FLC – it is a CCT domain-containing transcription factor and part of the type VI *CO-like* family of genes (Yan et al., 2004). Unlike, *FLC *in which chromatin-level suppression is the basis of memory,* VRN2 *suppression is not likely to be the primary event in memory for two reasons. One is that no changes in chromatin marks have been observed around the *VRN2 *locus during or after cold (Oliver et al., 2009). The other is that in *Brachypodium* *VRN2* mRNA levels are the same before and after cold exposure and this expression pattern is not consistent with *VRN2* acting as part of an evolutionarily conserved memory system in grasses (Ream et al., 2014).

*VRN1, *however, does exhibit cold-mediated chromatin changes. *VRN1 *is a repressor of *VRN2* and it is up-regulated in leaves by cold exposure and its increased expression is maintained in warm post-vernalization conditions (Yan et al., 2004; Sasani et al., 2009; Distelfeld and Dubcovsky, 2010; Chen and Dubcovsky, 2012). The activation of *VRN1* by cold is accompanied by a decrease in the repressive chromatin modification H3K27 methylation and an increase in activating H3K4 methylation in a presumed regulatory region of its first intron (Oliver et al., 2009, 2013)*. *The level of *VRN1* expression is proportional to the amount of cold experienced and thus correlates with the quantitative nature of the vernalization response in wheat, barley and *Brachypodium* (Yan et al., 2003; Sasani et al., 2009; Ream et al., 2014). Furthermore, the “henbane-like” behavior of certain *Brachypodium* accessions such as 29-1 enabled us to determine that increased *VRN1 *expression is maintained after cold exposure regardless of whether or not the plants are shifted to inductive photoperiods (**Figure 1D**). That *VRN1 *expression is maintained after cold exposure in SD – conditions in which *FT* is not expressed and flowering does not occur – demonstrates that vernalization causes a stable *VRN1* “on state” that is independent of *FT* expression (**Figures 1D,E**). This day length-independent stability of the on state of *VRN1* after cold exposure is consistent with a role for stable *VRN1* activation to contribute to the memory of the vernalized state in *Brachypodium*. That *VRN1* mRNA levels are higher in LD than in SD after vernalization is likely to be a result of FT enhancing *VRN1* expression as shown in other cereals (Yan et al., 2006; Sasani et al., 2009; Shimada et al., 2009; Distelfeld and Dubcovsky, 2010).

There are other candidates for genes that may have a role in the memory of winter in grasses. For example, Huan et al. (2013) recently identified several hundred genes in *Brachypodium* for which the expression patterns changed during cold and the changes are maintained after 7 days post cold. It will be interesting to determine which *Brachypodium* genes maintain vernalization-mediated expression changes during a “long-term memory test” of prolonged exposure to SD after vernalization. Recently *FLC-like* genes have been identified in monocots, but whether or not these *FLC-like* genes have a role in flowering in the grass lineage remains to be experimentally determined (Ruelens et al., 2013). There remains much to learn about the molecular basis of vernalization in temperate grasses.

## AUTHOR CONTRIBUTIONS

Daniel P. Woods, Richard M. Amasino, and Thomas S. Ream designed the experiments. Daniel P. Woods performed the experiments. Daniel P. Woods and Richard M. Amasino wrote and edited the manuscript.

## Conflict of Interest Statement

The authors declare that the research was conducted in the absence of any commercial or financial relationships that could be construed as a potential conflict of interest.
